# Supplementary material for: Factors associated with chronic opioid use after minimally invasive lung resections
Source: PLoS One. 2025 Jun 10;20(6):e0325354. doi: 10.1371/journal.pone.0325354 (PMC12151345; doi:10.1371/journal.pone.0325354)
Supplement: Attachment — Submitted filename: PLOS One First Review.docx [file pone.0325354.s001.docx]

Disclosure of Conflict of Interest: The Reviewer declares no personal, professional, or financial conflict of interest.

Confidentiality: Confidentiality was maintained in the review process of the manuscript.

Ethical Considerations: No ethical concerns,

**Manuscript Summary:**

- The manuscript entitled “Factors associated with chronic opioid use after minimally invasive lung resection” presents a study that aimed to identify factors associated with chronic opioid use after minimally invasive lung resections (MILR). 16.5% of patients used opioids at 30 days, 10.1% used at 60 days and 8.5% at 90 days; the variable identified as statistically significant predictors was morphine milligram equivalent (MMEs) of opioids on the day before discharge.
- The researchers can further expand on the discussion part, and reason out why other factors did not meet statistical significance.

**General Comments**:

1. Strengths: The study poses an important question on which factors are associated with chronic opioid use after a minimally invasive procedure like MILR.
2. Weaknesses: The study may not be powered enough to draw conclusions, as it is a single-center retrospective cohort study. Further, larger scale studies should be carried out.
3. Importance
   a. Does the study advance current knowledge or replicate previous work? YES
   b. Is the study clinically relevant? Will it impact patient care? Yes, the study is clinically relevant in the fields of anaesthesiology, critical care and pulmonary medicine.

**Specific Comments:**

4. **Title**: The title clearly and concisely addresses the work done.
5. **Abstract**:
   a. A structured abstract is presented.
6. **Introduction**
   a. The general problem or study intent is described.
   b. Relevant literatures were reviewed and referenced.
7. **Methods**
   a. IRB approval is stated.
   b. The methods are adequately explained and seem logical.
   c. The information presented in this section is essential, concise, and appropriate.
8. **Results**
   a. Data are presented in an understandable and logical sequence.
   b. Results for statistical evaluations and significance were included.
9. **Discussion**
   a. The findings are supported by the results.
   b. Findings are discussed in the context of existing knowledge and related publications, but further in-depth discussion, raising potential reasons for similarities and differences from previous studies is recommended. The gaps and limitations can further be discussed.
10. **References**
    a. The references are appropriate.
11. **Tables and figures**
    a. The titles are clear and appropriate
12. **Abbreviations** section is missing, and an exhaustive list should be included in the main manuscript.

**Confidential Comments to the Editor:**

**Recommendation**: The manuscript should be considered for acceptance after making some (minor) revisions, as detailed above.

**Thank you for your consideration.**

**Aman Edao Bime, MD, Assistant Professor of Anaesthesiology and Critical Care Medicine**
