# Supplementary material for: Factors associated with chronic opioid use after minimally invasive lung resections
Source: PLoS One. 2025 Jun 10;20(6):e0325354. doi: 10.1371/journal.pone.0325354 (PMC12151345; doi:10.1371/journal.pone.0325354)
Supplement: Attachment — Submitted filename: Response to Reviewers.docx [file pone.0325354.s003.docx]

Response to Reviewers

We thank the reviewers and the editor for their thoughtful feedback and the opportunity to revise our manuscript, *“Factors associated with chronic opioid use after minimally invasive lung resections.”* We have carefully addressed each comment below and made corresponding revisions to strengthen the manuscript. Our detailed responses follow:

**Response to Editor:**

1. Please ensure that your manuscript meets PLOS ONE's style requirements, including those for file naming.

**Response**: Thank you for the guidelines, we have formatted our submission to meet PLOS ONE’s style requirements.

2. We note that your Data Availability Statement is currently as follows: [All relevant data are within the manuscript and its Supporting Information files.]

Please confirm at this time whether or not your submission contains all raw data required to replicate the results of your study.

**Response**: We confirm that our submission contains all relevant data, it does not contain raw patient-level data since our institution’s protected health information is not something we can include. The Data Availability Statement has been updated to reflect this.

3. Please include a separate caption for each figure in your manuscript.

**Response**: Our 2 figures each have their own separate caption, appearing in the text at the first mention of each figure.

Please review your reference list to ensure that it is complete and correct. If you have cited papers that have been retracted, please include the rationale for doing so in the manuscript text, or remove these references and replace them with relevant current references. Any changes to the reference list should be mentioned in the rebuttal letter that accompanies your revised manuscript. If you need to cite a retracted article, indicate the article’s retracted status in the References list and also include a citation and full reference for the retraction notice.

**Response**: Our reference list was reviewed, there are no citation to retracted papers and no changes were made.

**Response to Reviewers:**

Reviewer #1: Comments:

- Title: The title clearly and concisely addresses the work done.
- Abstract: A structured abstract is presented.
- Introduction: The general problem or study intent is described.
  Relevant literatures were reviewed and referenced.
- Methods: IRB approval is stated. The methods are adequately explained and seem logical. The information presented in this section is essential, concise, and appropriate.
- Results: Data are presented in an understandable and logical sequence.

**Response to Reviewer #1:** Thank you for your positive and supportive comments. We appreciate the recognition of the clarity of the title, organization of the abstract, and the quality of the methodology and results presentation. No changes were made in response to this commentary.

Reviewer #2: I think this is an interesting topic and relevant. I would be interested in the authors discussing the patients pain scores, is there a relationship between the patients pain scores and the MME of the patient. After reading the manuscript I am questioning whether these patients are in more pain requiring more narcotics or are they just being prescribed more narcotics putting them at risk of chronic opioid use. It is probably beyond the scope of the study to answer that question but I think discussion would strengthen the manuscript.

**Response to Reviewer #2:** We appreciate your thoughtful comment and agree that the relationship between subjective pain and opioid usage is interesting and relevant. The possibility of patients receiving more pain medications due to more liberal prescription patterns should be explored in prospective studies. Unfortunately, in our analysis, routine pain scores were not reliably documented after discharge in a standardized format and therefore were not available for inclusion in the analysis. However, we have revised the discussion to address this important point, acknowledging the lack of pain score data as a limitation and discussing how this limits our ability to distinguish between pain-driven consumption versus prescription-driven risk. We have also emphasized the importance of future prospective research including pain scores to clarify this relationship.

We hope these revisions adequately address the reviewer’s concerns and improve the clarity and impact of our manuscript. We thank you again for your time and consideration

Sincerely,
*Hayley Reddington, MD, on behalf of all authors*
University of Massachusetts Chan Medical School
